# Impact of Ultra-High Pressure Homogenization on the Structural Properties of Egg Yolk Granule

**DOI:** 10.3390/foods11040512

**Published:** 2022-02-10

**Authors:** Romuald Gaillard, Alice Marciniak, Guillaume Brisson, Véronique Perreault, James D. House, Yves Pouliot, Alain Doyen

**Affiliations:** 1 Department of Food Science, Institute of Nutrition and Functional Foods (INAF), Université Laval, Quebec, QC G1V 0A6, Canada; romuald.gaillard.1@ulaval.ca (R.G.); alice.marciniak.1@ulaval.ca (A.M.); guillaume.brisson@fsaa.ulaval.ca (G.B.); veronique.perreault.5@ulaval.ca (V.P.); yves.pouliot@fsaa.ulaval.ca (Y.P.); 2 Department of Food and Human Nutritional Sciences, University of Manitoba, Winnipeg, MB R3T 2N2, Canada; james.house@umanitoba.ca

**Keywords:** egg yolk granule, ultra-high pressure homogenization, microstructure, protein aggregation, proteins

## Abstract

Ultra-high pressure homogenization (UHPH) is a promising method for destabilizing and potentially improving the techno-functionality of the egg yolk granule. This study’s objectives were to determine the impact of pressure level (50, 175 and 300 MPa) and number of passes (1 and 4) on the physico-chemical and structural properties of egg yolk granule and its subsequent fractions. UHPH induced restructuration of the granule through the formation of a large protein network, without impacting the proximate composition and protein profile in a single pass of up to 300 MPa. In addition, UHPH reduced the particle size distribution up to 175 MPa, to eventually form larger particles through enhanced protein–protein interactions at 300 MPa. Phosvitin, apovitellenin and apolipoprotein-B were specifically involved in these interactions. Overall, egg yolk granule remains highly stable during UHPH treatment. However, more investigations are needed to characterize the resulting protein network and to evaluate the techno-functional properties of UHPH-treated granule.

## 1. Introduction

Hen egg yolk is used in a wide variety of food products as an emulsifier and gelling agent due to its nutritional and techno-functional properties. It is particularly interesting because it contains proteins of high biological value, as well as other nutrients (phospholipids, vitamins, minerals, essential fatty acids) [1]. The protein fraction of egg yolk is composed of 68% low-density lipoproteins (LDLs), 16% high-density lipoproteins (HDLs), 10% globular proteins (livetins), 4% phosphoprotein (phosvitin), and 2% other minor proteins [2]. After centrifugation, egg yolk can be fractionated into two fractions called plasma (supernatant) and granule (pellet). The plasma is mainly composed of LDLs (85%) and livetin (15%), whereas the granule consists of HDLs (70%), phosvitin (16%) and LDLs (12%). The granule has interesting nutritional properties related to its low cholesterol (LDLs) content [3,4,5] and high 5-methyl-tetrahydro-folate (5-MTHF) concentration, compared to the whole egg yolk [6]. Ultimately, destabilizing the egg yolk granule could improve the accessibility of its nutritional and bioactive compounds and eventually improve the digestibility and functionality of the product [7]. Structurally, the granule consists of a circular structure formed by non-soluble complexes involving HDL and phosvitin stabilized by phosphocalcic bridges [8]. The large number of phosphocalcic bridges gives the granule a very compact and poorly hydrated structure, leading to its high resistance to thermal treatment, ultrasound and enzymatic hydrolysis [9,10].

Different strategies have been applied to destabilize the egg yolk granule. Usually, this involves increasing the ionic strength, which induces the disruption of phosphocalcic bridges and improves the granule solubility [7,8]. This high solubility improves the emulsion stability of the granule compared to the egg yolk and plasma [7]. More recently, high hydrostatic pressure (HHP) (400 and 600 MPa, for 5 min) applied to the granule was sufficient to destabilize its structure, probably by disrupting the phosphocalcic bridges between phosvitin and HDLs [11,12]. After centrifugation of the isostatically pressured granule, the recovered insoluble fraction was enriched in folic acid and phosvitin. Consequently, the use of high pressure shows great potential to generate new ingredients with interesting nutritional and functional properties for many industrial applications such as use as a low-cholesterol emulsifying agent in mayonnaise [11,12,13,14,15].

The ultra-high pressure homogenization (UHPH) system, with higher pressure levels of up to 400 MPa, is an emerging technology used to modify the structure of food matrices. Contrary to the isostatic HHP process, UHPH is a dynamic pressure process inducing turbulence, high shear, cavitation, and temperature to help reduce particle size distributions, allowing emulsion stability to be enhanced [16,17]. UHPH also induced modification of protein structures through unfolding of the quaternary and tertiary structure, and generation of inter- and intra-molecular interactions [16,18,19,20]. However, these modifications depend on the matrices and pressure parameters applied, such as pressure level, process temperature and the number of passes. These particular modifications of protein structure and interactions induced by UHPH were studied to improve the techno-functional properties of proteins [21,22], such as emulsifying [23,24,25,26] and foaming [27,28,29,30,31,32].

To the best of our knowledge, limited data are available regarding the impact of UHPH on granule destabilization. Only Sirvente et al. [10] demonstrated that conventional high pressure homogenization (0.3 to 20 MPa) had little effect on granule destabilization and its techno-functional properties. Thus, the use of UHPH for the destabilization of the compact granule structure represents a promising technology for generating a high value-added egg fraction with interesting functional and nutritional properties for the food industry [28,31]. Consequently, this project aims to study the impact of the pressure level (50, 175 and 300 MPa) and number of passes (1 and 4) on the structural properties of egg yolk granule and its subsequent fractions (plasma and granule from pressure-treated granule).

## 2. Materials and Methods

### 2.1. Preparation of Egg Yolk Granules

Fresh hen eggs were purchased from a local supermarket and stored at 4 °C until preparation according to the protocol described by Naderi et al. [6]. Briefly, fresh hen eggs were manually broken, and the albumen was discarded. Albumen residues were eliminated from yolk by absorption on filter paper (Whatman, MA, USA). The vitelline membrane of yolk was removed with tweezers. Egg yolks were diluted in distilled water (1:1 *w/w*) and centrifuged at 10,000× *g* for 45 min at 4 °C for the recovery of plasma (supernatant) and granule (pellet) fractions. The granule fraction was prefrozen at −30 °C and freeze-dried (pressure of 27 Pa and plate temperature of 20 °C) for 48 h. The freeze-dried granule was kept frozen under vacuum until analysis and treatment.

### 2.2. Ultra-High Pressure Homogenization

**Figure 1:**
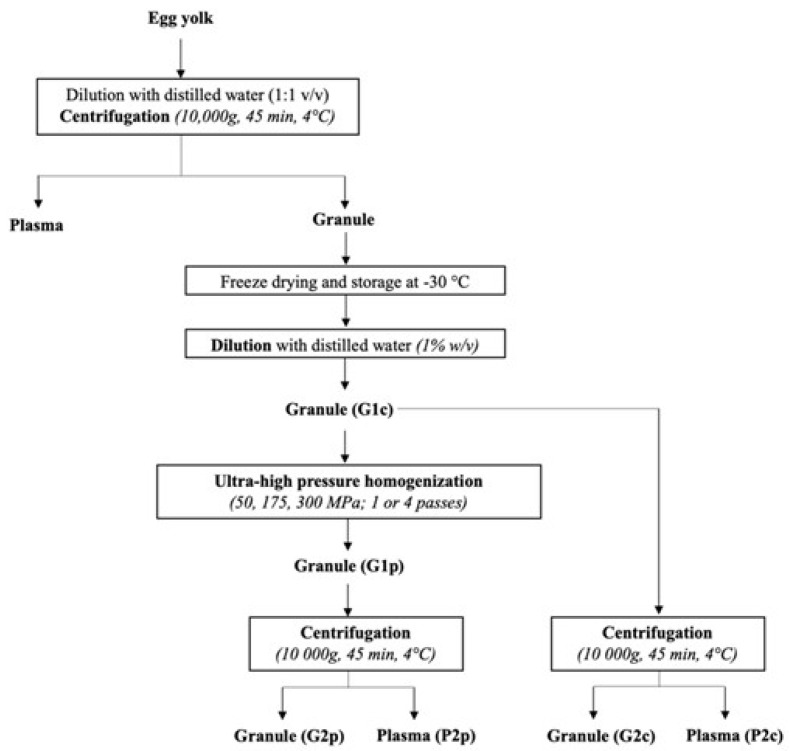
Experimental design of the production of the different fractions from egg yolk granule after ultra-high-pressure homogenization treatments. G1c: initial control granule, G1p: initial pressure-treated granule, G2p: granule from initial pressure-treated granule, P2p: plasma from initial pressure-treated granule, G2c: control granule from initial control granule, P2c: control plasma from initial control granule.

The freeze-dried granule was solubilized in distilled water (1% *w*/*v*) overnight at 4 °C under constant agitation to generate the initial granule solution (G1c). Immediately after solubilization, the G1c was pressure-treated by single or multiple-pass (1 to 4) ultra-high pressure homogenization (Nano DeBEE laboratory homogenizer, Bee International, South Easton, MA, USA) at 50, 175 and 300 MPa at a flow rate of 4 L/h. While it is estimated that the temperature of the sample increased by around 16 to 25 °C per 100 Mpa [31,33,34], the system was equipped with a cooling system allowing a fast temperature decrease after UHPH treatment. The resulting emulsions obtained after pressurization of G1c were labeled G1p. After UHPH treatments, 1 mL of sodium azide solution (0.02% *w*/*v*) was added to 500 mL of sample for preservation. Next, pressure-treated granule samples (G1p) were centrifuged at 10,000× *g* for 45 min at 4 °C to generate a second granule (G2p) and plasma (P2p). Control granule (G2c) and plasma (P2c) were generated following the same experimental design without pressurization treatment (Figure 1). Secondary granules (G2p and G2c) were resuspended in 10 mL of distilled water for native PAGE analysis. Finally, initial granules (G1p and G1c), secondary granules (G2p and G2c) and plasma (P2p and P2c) were prefrozen at −30 °C and freeze-dried (pressure of 20 Pa and plate temperature of 20 °C) for 48 h before subsequent analysis.

### 2.3. Proximate Composition

Dry matter and ash content of the initial control granule (G1c), pressure-treated granule (G1p), granule from pressure-treated granule (G2p) and control granule (G2c) fractions were determined according to the Association of Official Agricultural Chemists (AOAC) 923.03 (dry matter) and 925.09 (ash) methods. The crude protein content of G1c, G2c, G1p, G2p, control plasma (P2c) and plasma originating from pressure-treated granule (P2p) was obtained by the Dumas method (LECO FP-528, Model 601-500, LECO, St. Joseph, MI, USA) using a protein-to-nitrogen conversion factor of 6.25 [6]. The lipid content of G1c, G1p, G2p and G2c fractions was determined by the Mojonnier method (AOAC International 925.32). Phosphorus and iron contents of G1c, G1p, G2p and G2c fractions were obtained by inductively coupled plasma optical emission spectroscopy (ICP-OES) (Agilent 5110 ICP-OES, Agilent Technologies, Inc., Santa Clara, CA, USA) according to the AOAC 923.03 and 925.09 methods. Dry matter, lipid, ash and mineral composition were not determined for P2c and P2p due to limited quantities of those fractions.

### 2.4. Particle Size Distribution

The distribution of particle sizes of G1c and G1p was determined on liquid samples using a laser diffraction system (Malvern Mastersizer 3000, Malvern Instrument Ltd., Worcestershire, UK), and the data were analyzed with the software Mastersizer V.3.72. Samples were analyzed without dilution. The sample dispersion particle type was set to non-spherical particle mode and the refractive index was set to 1.45, whereas the dispersant phase’s (deionized water) refractive index was set to 1.33. The sample was added to the cell up to an obscuration rate between 8–13%. The mean diameter expressed as volume mean (De Brouckere mean diameter) was expressed as D_[4,3]_. All conditions were analyzed in duplicate with 3 measures for each duplicate.

### 2.5. Microstructure of Egg Yolk Granules and Fractions

The microstructure of control and pressure-treated granule and plasma samples was visualized by transmission electron microscopy (TEM) (JEOL JEM-1230 TEM, Tokyo, Japan) operating at 80 kV and at a magnification factor of 3K. Granule fractions (G1c, G1p, G2c and G2p) were diluted to 1:200 for obtaining good quality images, whereas plasma fractions (P2c and P2p) were used undiluted. A droplet of each sample was mixed with 3% (*w*/*v*) uranyl acetate and placed onto a copper grid/carbon film for 1 min. The excess was removed, and the grid was air-dried. A Gatan camera ultrascan US1000SP1 (Gatan, Inc., Pleasanton, CA, USA) was used for image capture.

### 2.6. Protein Profiles

Protein profiles of control and pressure-treated samples were obtained by native and sodium dodecyl sulphate (SDS)-polyacrylamide gel electrophoresis (PAGE) under reducing conditions according to the protocol of Naderi et al. [6]. First, native PAGE was carried out with fresh liquid samples. For control and pressure-treated granule fractions, 25 µL of diluted sample (1:4 *v*/*v*) was mixed with 25 µL of native sample buffer (62.5 mM Tris-HCl, 40% glycerol, 0.01% bromophenol blue—Bio-Rad, Mississauga, ON, Canada), and 10 µL of each sample was loaded into the well. For control and pressure-treated plasma fractions, 50 µL of sample and 20 µL of native sample buffer were mixed and a final volume of 20 µL was loaded into each well. For SDS PAGE under reducing conditions, a 1% (*w/v*) protein solution was prepared for each freeze-dried granule and plasma fraction, and 200 µL of that solution was diluted into 800 µL of deionized water. Samples G2c and G2p were sonicated for 30 min to improve solubility. A volume of 25 µL of sample buffer (5% 2-mercaptoethanol, 95% Laemmli buffer—Bio-Rad, Mississauga, ON, Canada) was added to 25 µL of each diluted sample. Samples were boiled for 10 min and cooled on ice before loading 10 µL into the gel wells. Electrophoresis was performed using 4–20% TGX Stain-Free polyacrylamide gel (Bio-Rad, Mississauga, ON, Canada) and run at 30 mA per gel for a total of 35 min at room temperature. Gels were stained with 0.1 M aluminum nitrate and destained with a solution of 40% *v*/*v* methanol, 10% *v*/*v* acetic acid and 50% *v*/*v* distilled water. The molecular weights of migrated proteins were estimated using molecular weight markers (Precision Plus Protein™ 161-0373 All Blue Prestained Protein Standards, Bio-Rad, Mississauga, ON, Canada). Images of the gels were captured using the ChemiDoc™ MP Imaging System (Bio-Rad Laboratories Ltd., Mississauga, ON, Canada).

### 2.7. Proteomics Analysis

Protein digestion and mass spectrometry analyses were performed by the Proteomics Platform of the Research Center of the Centre Hospitalier Universitaire (CHU) of Quebec (Quebec City, QC, Canada). Specific bands corresponding to high molecular weight aggregates from native gels were excised for G1c and G1p at 300 MPa with 1 and 4 passes and washed with MilliQ water. Tryptic digestion products for each sample were separated by online reversed-phase (RP) nanoscale capillary liquid chromatography (nanoLC) and analyzed by electrospray mass spectrometry (ES MS/MS). Data obtained by mass spectrometry were analyzed using Mascot (Matrix Science, London, UK; version 2.5.1) as described by Duffuler et al. [13]. The Uniprot Gallus 2020 database and Scaffold software (version Scaffold_4.11.1, Proteome Software Inc., Portland, OR) were used for peptide and protein identification. The false discovery rates (FDR) and the minimum number of peptides occurrence were set to <1.0% and 2, respectively.

### 2.8. Statistical Analysis

Data analyses were carried out using Statistical Analysis System (SAS), SAS^®^ Studio software (Copyright © 2020 SAS Institute Inc., Cary, NC, USA.) and three replicates were performed for each experiment. The proximate compositions of the fractions were analyzed by one-way analysis of variance (ANOVA) and the Tukey test (α = 0.05) for multiple comparisons. The particle size distribution and the mean diameter expressed as volume mean, D_[4,3]_, were analyzed by multifactorial ANOVA. A 95% confidence interval (*p* < 0.05) was used for all tests. Data were expressed as mean ± standard deviation (SD).

## 3. Results

### 3.1. Proximate Composition of Control and Pressure-Treated Egg Yolk Fractions

Table 1 shows the proximate composition of control and pressure-treated fractions for each pressurization level (50, 175 and 300 MPa) and number of passes (1 and 4). No differences (*p* > 0.05) were observed regarding the content of dry matter and all components (protein, lipid, ash, phosphorus and iron) between control and pressure-treated fractions (granule or plasma), regardless of the pressure applied (50, 175 and 300 MPa) and the number of passes (1 and 4).

### 3.2. Effect of UHPH Treatments on Particle Size Distribution

**Figure 2:**
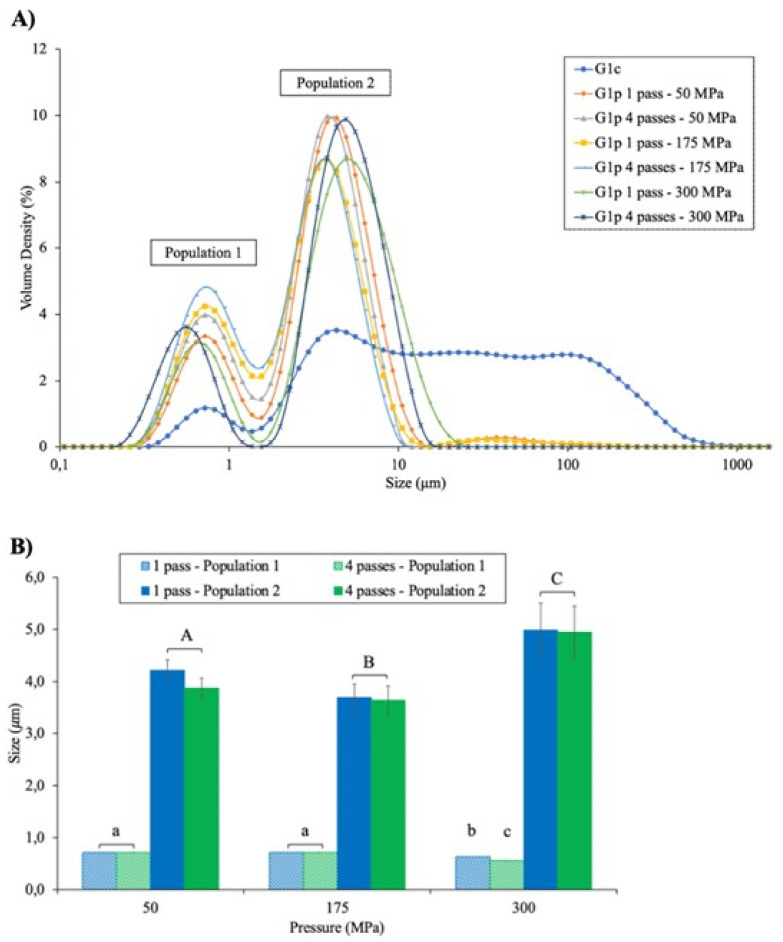
Particle size distribution of initial (G1c) and pressure-treated granule (G1p) at 50, 175 and 300 MPa with 1 and 4 passes (**A**), and factorial effect of pressure level (50, 175 and 300 MPa) and number of passes (1—blue and 4—green) on the two populations (1—hatched and 2—filled) (**B**). Different letters, a–c for population 1 and A–C for population 2, indicate significant differences (*p* < 0.05) due to interaction between pressure and passes and solely due to the pressure level (Tukey test, α = 0.05, *n* = 3).

Figure 2 shows the particle size distribution of initial control (G1c) and pressure-treated granule (G1p) at 50, 175 and 300 MPa with 1 and 4 passes (Figure 2A), and the factorial effect of pressure level and number of passes (1 and 4) on the two main populations (Figure 2B). The two main heterogenous populations were distinguishable in the non-treated fraction (G1c), with a particle size between 0.33 and 1.36 µm for population 1 and between 1.76 and 625 µm for population 2 (Figure 2A). The application of UHPH treatments decreased the particle size of the initial control granule (G1c) drastically. Particles above about 30 µm that were initially present were reduced to two main populations: population 1 ranging from 0.25 to 1.55 µm and population 2 ranging from 1.76 to 15.4 µm.

To understand the impact of pressure level (50, 175 and 300 MPa) and number of passes (1 and 4) on the size of each population, a multiple factorial analysis was performed for all G1p fractions (Figure 2B). The figure represents the size corresponding to the highest intensity for each population (1—hatched and 2—filled) as a function of pressure (50, 175 and 300 MPa) and passes (1—blue and 4—green). Population 1 had strong interactions between both pressure and passes (*p* < 0.0001). While no effect was observed for the number of passes (1 or 4) at 50 and 175 MPa, at 300 MPa, population 1′s particle sizes for 1 (0.63 µm) and 4 passes (0.56 µm) were lower than those obtained at 50 and 175 MPa (mean of 0.72 µm). In contrast, for population 2, the impact of UHPH was solely due to the pressure level (*p* < 0.0001). Indeed, the particle size decreased from 4.04 to 3.66 µm for 50 and 175 MPa, and finally increased from 3.66 to 4.96 µm for 175 and 300 MPa.

**Figure 3:**
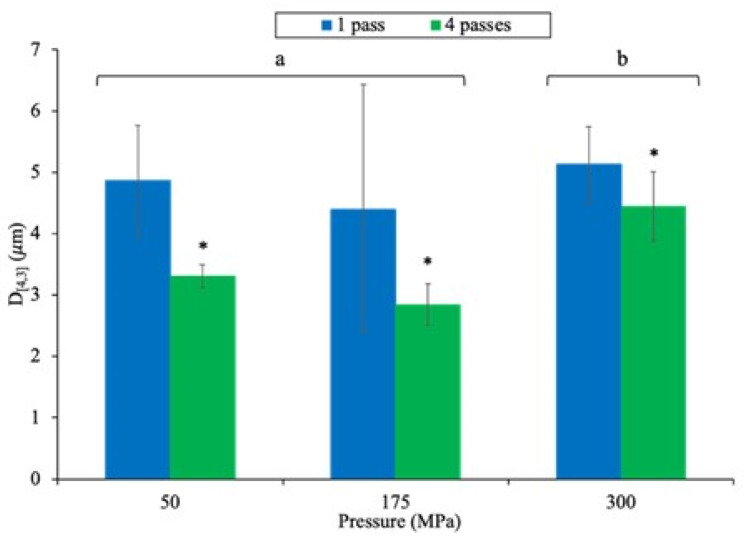
Impact of pressure level (50, 175 and 300 MPa) and number of passes (1—blue and 4—green) on the mean diameter expressed as volume (D_[4,3]_) of pressure-treated granule (G1p). Data with different letters (a-b) and * are significantly different (*p* < 0.05), due to a simple effect of pressure and passes (Tukey test, α = 0.05, *n* = 3).

Figure 3 represents the mean diameter expressed as volume (D_[4,3]_), which reflects the sizes of particles that constituted the majority of the sample volume of pressure-treated granule (G1p) as a function of pressure (50, 175 and 300 MPa) and number of passes (1 and 4). The D_[4,3]_ value of the initial granule (G1c—data not shown) was higher (*p* < 0.0001) than that of pressure-treated granules, with average values of 58.5 ± 43.2 µm for G1c and 4.2 ± 0.8 µm for all G1p, which corresponded to a decrease of approximately 93%. Within UHPH treatments, the multiple factorial analyses indicated a simple effect of pressure (*p* < 0.0001) and passes (*p* < 0.0001) without interaction between these two parameters (Figure 3). Globally, regardless of the pressure level (50–300 MPa), UHPH at 1 pass increased the D_[4,3]_ value compared to that in samples pressurized with 4 passes (*p* < 0.0001), with average values of 4.8 ± 1.2 µm for G1p at 1 pass and 3.5 ± 0.4 µm for G1p at 4 passes. In addition, regardless of the number of passes, pressure treatment at 300 MPa induced higher D_[4,3]_ compared to pressures of 50 and 175 MPa, with values of 4.8 ± 0.6 µm (average of G1p at 300 MPa at 1 and 4 passes) and 3.9 ± 0.9 µm (average of G1p at 50 and 175 MPa at 1 and 4 passes).

### 3.3. Microstructural Modifications Following UHPH Treatments

**Figure 4:**
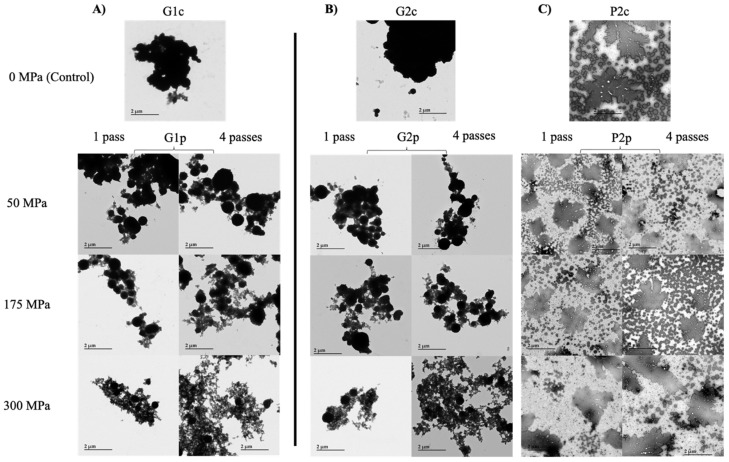
Microstructures of (**A**) initial control granule (G1c) and pressure-treated granule (G1p), (**B**) control granule from initial control granule (G2c) and granule from pressure-treated granule (G2p), and (**C**) control plasma from initial control granule (P2c) and plasma from pressure-treated granule (P2p) observed by transmission electron microscopy with a magnification of 3K.

Figure 4 shows the changes in microstructure associated with the initial granule (G1c) before and after UHPH treatment (G1p—Figure 4A), and their respective secondary granules (G2c and G2p—Figure 4B) and plasmas (P2c and P2p—Figure 4C) using TEM. First, the initial granule (G1c) appeared as large and compact (dark) aggregates with diameters close to 4 µm, whereas a change in the morphology of the particles was observed after UHPH treatment, correlating with the increase in pressure and number of passes (Figure 4A). Specifically, the particles observed for the pressure-treated granule (G1p) at 300 MPa with 4 passes formed a diffuse and thin network compared to the particle shape in the initial control granule (G1c). Second, the control granule from initial granule (G2c) and granule from pressure-treated granule (G2p) (Figure 4B) showed microstructure and effects of pressure and number of passes similar to those observed for G1c and G1p. Last, the microstructure of control plasma from initial granule (P2c) and plasma from pressure-treated granule (P2p) (Figure 4C) was drastically different from that of granules (G1 and G2) (Figure 4A,B). Indeed, TEM images show very particular leaf-like structures but none of the network previously observed in G1p and G2p. It should be noted that no major differences, regardless of pressurization level (50–300) and number of passes (1–4), were observed between plasma samples. Compared with the controls (G1c and G2c), UHPH treatments caused major changes in the microstructure of the particles in the granule fractions that correlated with the increases in pressure (50–300 MPa) and number of passes (1–4). UHPH treatments reduced the particle size and changed the microstructure of the complex, thus forming a thin and highly diffuse network when subject to the most severe parameters (300 MPa, 4 passes).

### 3.4. Effect of UHPH Treatments on Protein Profiles

**Figure 5:**
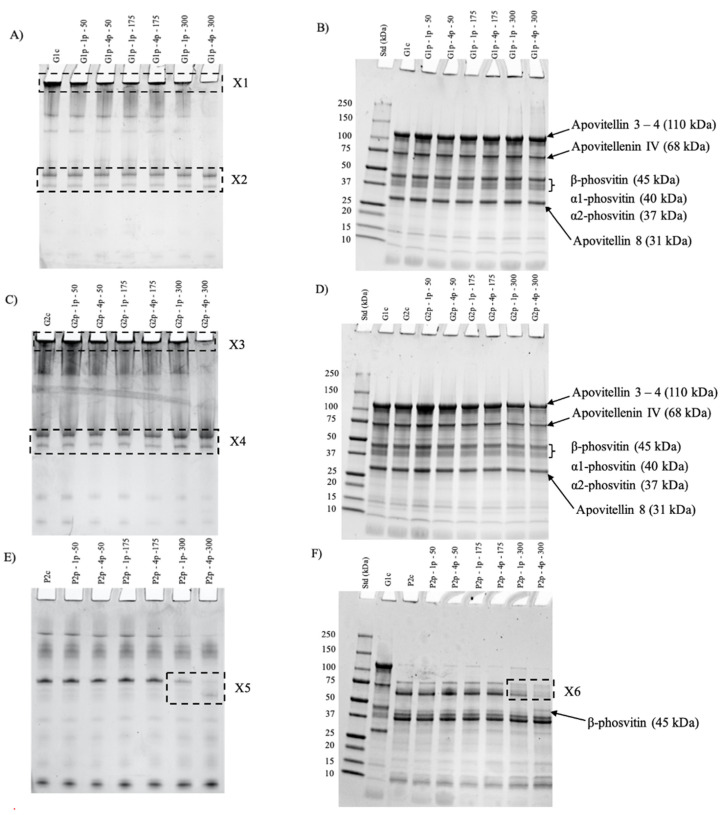
Native (**A**,**C**,**E**) and reduced (**B**,**D**,**F**) SDS PAGE of initial granule (G1c) and pressure-treated granule (G1p) (**A**,**B**); control granule from initial granule (G2c) and granule from pressure-treated granule (G2p) (**C**,**D**); and control plasma from initial granule (P2c) and plasma from pressure-treated granule (P2p) (**E**,**F**).

Figure 5 shows the native (Figure 5A,C,E) and reduced (Figure 5B,D,F) SDS PAGE of G1c and G1p (Figure 5A,B), G2c and G2p (Figure 5C,D) and P2c and P2p (Figure 5E,F) fractions. Protein profiles from native PAGE of the initial (G1c) and pressure-treated granule (G1p) (Figure 5A) were similar except that the intensity of the band in the loading well (X1) decreased as a function of the increase in pressure level, mainly at 300 MPa and passes. Two other bands (X2) with similar intensities were detected in native PAGE gels through all pressure levels and numbers of passes. Native PAGE of the granule obtained after centrifugation of initial granule (G2c) and pressure-treated granule (G2p) (Figure 5C) showed that the intensity in the loading well (X3) decreased as a function of UHPH treatments, mainly for 300 MPa with 4 passes, as observed for G1c and G1p (Figure 5A). Similarly to G1c and G1p, no differences were observed in the protein profile (X4) for all G2c and G2p fractions (Figure 5B). Native PAGE of plasma (P2c and P2p) fractions (Figure 5E) showed no remarkable modifications in the protein profiles between P2c and P2p at 50 and 175 MPa, regardless of the number of passes. However, at 300 MPa, differences in the protein profiles (X5) were observed after 1 and 4 passes.

The protein profiles of control and pressure-treated granule fractions (Figure 5B,D), obtained from SDS PAGE under reducing conditions showed 6 major protein bands from 31 kDa to 110 kDa that were also identified by Guilmineau et al. [35], Naderi et al. [5] and [12]. Their intensity and distribution remained similar, regardless of the treatment and granule sample. The protein profiles of plasma from pressure-treated granule (P2p) (Figure 5F) obtained from SDS PAGE under reducing conditions were similar to those of the control for samples at 50 and 175 MPa with 1 or 4 passes. However, and as observed in native PAGE (Figure 5E), at 300 MPa, the intensity of the protein bands at 68 and 78 kDa (X6) decreased as a function of the number of passes. In addition, for P2p at 300 MPa and 4 passes, a band with a molecular weight of 45 kDa appeared that was not present for other treatments and control (P2c).

### 3.5. Characterization of Egg Yolk Proteins by Proteomics Analysis

The band observed in the wells of native PAGE of initial granule (G1c) and pressure-treated granule (G1p) at 300 MPa and 1 pass and 4 passes were analyzed by LC-MS/MS, and the results are presented in Table 2. The total spectrum count (TSC) value corresponds to the total number of spectra identified for a protein and is a semi-quantitative measure for a given protein abundance in proteomic studies [36], and the coverage percentage corresponds to the percentage of the protein sequence represented by the detected peptides in the dataset [37]. Globally, the composition of all G1c and G1p fractions at 300 MPa and 1 pass and G1p at 300 MPa and 4 passes were identical. A total of eight specific proteins from egg yolk granule were identified for all samples, with five predominant proteins with molecular weight ranging from 12 to 523 kDa. These proteins were identified as phosvitin from vitellogenin (VTG)-2 (#1—205 kDa), VTG-1 (#2—211 kDa) and VTG-3 (#3—187 kDa), apolipoprotein-B (#4—523 kDa), and apovitellenin-1 (#5—12 kDa). For these proteins, the coverage percentage and TSC ranged from 41% to 73% and from 17 to 1235, respectively, for all conditions (Table 2).

## 4. Discussion

The aim of the current work was to investigate the destabilization of egg yolk granule through UHPH treatment. Overall, the approximate compositions of granule and the resulting granule and plasma fractions were not modified following UHPH treatments. However, the application of UHPH decreased the average particle size of the initial granule (G1c). Additionally, the mean diameter of particles expressed as volume (D_[4,3]_) increased for pressures above 300 MPa, and to a greater extent, when treated with 4 passes. These observations correlated with a change in the microstructure of the granule through the formation of a diffuse and thin network. Regardless of the treatment (number of passes or pressure level), phosvitin, apovitellenin and apolipoprotein-B were involved in the formation of these structures.

### 4.1. Impact of UHPH Treatments on Particle Size Distribution of Initial (G1c) and Pressure-Treated Granule (G1p)

In the granule, HDL, LDL and phosvitin are associated with phosphocalcic bridges. Depending on the conditions (pH and ionic strength), the HDL–phosvitin complex can form soluble micelles with particle sizes ranging from 100 to 200 nm [1]. These structures could correspond to the large population observed in granule samples without application of UHPH treatments (Figure 2A—Population 2). However, after pressurization treatments, a reduction in particle size distribution as well as an increase in the volume density and a decrease in the mean diameter D_[4,3]_ values were observed, compared to the control. These observations were noted regardless of the pressure level and number of passes, indicating that the global UHPH treatment-related mechanical force and high temperatures encountered in the valve could disperse granule aggregates into small particles. These results agree with those obtained by numerous authors using model oil-in-water emulsions [23] and protein-stabilized emulsions [19,38,39,40]. Our observations were also similar to those of Naderi et al. [11] using ultrasound treatment, which were explained by the cavitation and high shear forces inducing disintegration of the granule into small particles of HDL-phosvitin [11]. In addition, and contrary to the control granule sample, bimodal and polydisperse distributions were observed after UHPH treatments (Figure 2A), as observed by Sirvente et al. [10] after mechanical treatment of yolk granule by conventional high-pressure homogenization (0.3, 10 and 20 MPa). According to Chang et al. [41] and Causeret et al. [8], the smallest particle size population could correspond to a spherical complex of non-soluble HDL-phosvitin linked by phosphocalcic bridges where the largest particle size population was probably granule aggregates [1].

For the smallest population of particles (Figure 2B—hatched), we observed a strong interaction between both pressure and passes on particle size reduction, with the largest decrease observed at 300 MPa and 4 passes. Increasing the homogenization pressure progressively reduced the size particle, while distributions became narrower, potentially due to the reduction in size of HDL caused by homogenization partially disrupting the granules [42], as observed for oil-in water emulsions prepared with egg yolk. Similar results were obtained after application of UHPH to milk fat globule membrane (MFGM), which is mainly composed of phospholipids (like yolk granule), in the presence of milk protein [43]. Simultaneously, increasing the number of passes reduced the sizes of proteins due to the unfolding phenomenon [44]. Increasing the number of passes could also induce greater disruption of the HDL–phosvitin complex and decrease its particle size (Figure 2B), as observed in oil-in-water emulsions composed of whey protein isolate and flaxseed oil [45]. On the other hand, the volume density of the second population observed in Figure 2A was greater than that of the first population. UHPH treatments are known to modify the conformation of globular proteins, which tend to unfold and aggregate, leading to protein–protein interactions [19,21,46]. Consequently, population 2 could be made up of yolk protein aggregates generated by the combination of mechanical homogenization and shear-induced temperature effects throughout UHPH treatment. A detailed analysis of population 2 (Figure 2B—filled) showed that only pressure level had an impact on particle size distribution. More specifically, the particle size decreased between 50 and 175 MPa to eventually increase at 300 MPa. The same tendency was shown by the D_[4,3]_ values (Figure 3). These observations could be the result of increased interaction and subsequent re-coalescence of smaller fat and protein particles, as observed after microfluidization treatment of egg yolk [47]. As indicated previously, the higher the pressure, the smaller the particle size due to a drastic disruption of granule structure, specifically HDL–phosvitin complex. As a result, the protein–protein aggregation phenomenon could also occur at higher pressure levels.

### 4.2. Impact of UHPH Treatments on the Protein Profile, Composition and Microstructure of Egg Yolk Fractions

Overall, the results showed a drastic reorganization of the granule’s microstructure with the severity of UHPH treatment (up to 300 MPa—4 passes) but little impact on its protein composition. The application of UHPH treatment up to 175 MPa induced the large particles present in the granule to be disrupted into smaller particles, as observed by TEM analysis (Figure 4A,B). The plasma fraction, however, showed irregular leaf-like structures comparable to the one noticed by Staneva et al. [48] with mixture of egg phosphatidylcholine, sphingomyelin and ceramide (Figure 4C). Observations on granule fractions correlated with the particle size measurements discussed previously and were supported by the literature on conventional high-pressure homogenization [10,49,50]. The mechanical forces involved during high pressure homogenization disrupt the quaternary structure of proteins and eventually lead to structural changes through protein unfolding [51]. In addition, when samples were pressurized at 300 MPa, TEM analysis showed the formation of a thin and diffuse network (Figure 4A,B). These results agree with the findings of Floury et al. [19], who observed a gel-like network structure in UHPH-treated soy protein-stabilized oil-in-water emulsions at pressures above 250 MPa. The increase in pressure causes the temperature within the valve to increase, thus increasing the strength of the hydrophobic bonds. The resulting protein–protein interactions can be enhanced to eventually form a gel-like network [19]. In addition, as observed by Marco-Molés et al. [24] with egg/dairy oil-in-water emulsions, the use of pressure up to a critical level (300 MPa in our study) could induce destabilization of an emulsion through the loss of the natural emulsifier barrier, thus potentially increasing protein–protein interactions.

Proteomic analysis (Table 2) and gel electrophoresis (Figure 5B,D) showed that the protein composition of pressure-induced structures was similar regardless of the treatment, and specifically composed of apovitellenin, apolipoprotein and phosvitin. This also indicates the high resistance of granule proteins to UHPH treatment compared to HHP treatment. These observations agree with the literature on heat treatment of egg yolk, which demonstrates very little impact of the treatment on the granule proteins vs. plasma proteins [52,53]. The high resistance of phosphocalcic bridges toward heat could explain granule stability, especially the HDL–phosvitin complex, against UHPH treatment [1]. In this work, the application of UHPH up to 300 MPa—1 pass induced the disintegration of the initial granule structure into smaller particles composed of HDL–phosvitin complexes. However, when treated at 300 MPa—4 passes, the combination of pressure and high temperature, as well as mechanical forces, enhanced protein–protein interactions, and the formation of a protein network. It also caused a very slight dissociation of the β-phosvitin from HDL complexes, leading to the release of a small portion of the β-phosvitin into the soluble fractions (plasma) (Figure 5F). These observations differ from the literature on the application of HHP to egg yolk granule. Indeed, Naderi et al. [11] showed HHP treatment (600 MPa, 5 min) disrupted the egg yolk granule structure and induced a specific and major transfer of phosvitin and folate into the plasma fraction.

## 5. Conclusions

Ultra-high pressure homogenization (50–300 MPa for 1–4 passes) was used as a pre-treatment technology to destabilize the native compact structure of egg yolk granule. This study demonstrates that increasing the pressure to 300 MPa and the number of passes to 4 induces structural modification of the granule by decreasing the particle size and increasing protein–protein interactions to eventually form a protein network. Despite the harsh conditions generated during the most severe treatment (300 MPa at 4 passes), the phosvitin–HDL complex showed very high resistance, thus not significantly impacting the composition of the network consisting of apovitellenin, apolipoprotein and phosvitin. However, as this work was preliminary to further investigations, the extent of protein unfolding and the nature of the interactions involved during formation of a protein network by UHPH treatments of egg yolk granule remain to be investigated. Finally, the next step will be to understand the impact of structural modification on the functional properties of egg yolk granules treated by UHPH.

## Figures and Tables

**Table 1:** Composition of control and pressure-treated fractions of egg yolk.

Fraction	Dry Matter	Protein	Lipid	Ash	Phosphorus (P)	Iron (Fe)
	(% *w*/*w*, Dry Basis)					(×10^−2^)
G1c (control)	96.1 ± 1.0 ^a^*	64.6 ± 0.7 ^a^	39.7 ± 11.2 ^a^	6.5 ± 0.2 ^a^	1.1 ± 0.5 ^a^	2.4 ± 1.2 ^a^
G1p (50, 175 and 300 MPa for 1 and 4 passes)	96.5 ± 2.2 ^a^	62.7 ± 0.8 ^a^	29.7 ± 5.0 ^a^	6.4 ± 0.2 ^a^	1.0 ± 0.4 ^a^	1.5 ± 0.8 ^a^
G2c (control)	99.0 ± 0.6 ^a^	63.6 ± 1.2 ^a^	37.3 ± 6.4 ^a^	5.4 ± 0.6 ^b^	0.5 ± 0.2 ^a^	1.9 ± 0.7 ^a^
G2p (50, 175 and 300 MPa for 1 and 4 passes)	99.1 ± 0.4 ^a^	62.8 ± 2.1 ^a^	42.0 ± 8.6 ^a^	5.5 ± 0.2 ^b^	0.5 ± 0.2 ^a^	1.5 ± 0.4 ^a^
P2c (control)	N.D.	50.0 ± 10.7 ^b^	N.D.	N.D.	N.D.	N.D.
P2p (50, 175 and 300 MPa for 1 and 4 passes)	N.D.	50.1 ± 3.6 ^b^	N.D.	N.D.	N.D.	N.D.

* Mean value ± standard deviation. Data with different letters (a–b) within each column are significantly different at *p* < 0.05 (Tukey test, α = 0.05, *n* = 3). G1c: initial granule, G1p: pressure-treated granule, G2c: control granule from initial granule, G2p: granule from pressure-treated granule, P2c: control plasma from initial granule, P2p: plasma from pressure-treated granule. N.D.: not determined.

**Table 2:** Proteomic analysis of native PAGE wells of initial granule (G1c), pressure-treated granule (G1p) at 300 MPa—1 pass and pressure-treated granule (G1p) at 300 MPa—4 passes.

# Protein	Identified Proteins	Molecular Weight (kDa)	UniProt ID	% Coverage			Total Spectrum Count (TSC)		
				G1c	300 MPa 1 Pass	300 MPa 4 Passes	G1c	300 MPa 1 Pass	300 MPa 4 Passes
**1**	Phosvitin OS = *Gallus gallus* OX = 9031 GN = VTG2 PE = 4 SV = 1	205	F1NFL6_CHICK	63	59	73	1235	660	1227
**2**	Phosvitin OS = *Gallus gallus* OX = 9031 GN = VTG1 PE = 4 SV = 1	211	A0A1D5NUW2_CHICK	56	48	60	523	341	608
**3**	Phosvitin OS = *Gallus gallus* OX = 9031 GN = VTG3 PE = 4 SV = 1	187	A0A3Q2U347_CHICK	55	49	63	267	197	309
**4**	Apolipoprotein B OS = *Gallus gallus* OX = 9031 GN = APOB PE = 4 SV = 2	523	F1NV02_CHICK	51	48	59	374	401	571
**5**	Apovitellenin-1 OS = *Gallus gallus* OX = 9031 PE = 1 SV = 1	12	APOV1_CHICK	65	41	63	34	17	36
**6**	Albumin OS = *Gallus gallus* OX = 9031 GN = ALB PE = 4 SV = 2	64	A0A1D5NW68_CHICK	9	68	82	8	50	86
**7**	Ovotransferrin OS = *Gallus gallus* OX = 9031 GN = TF PE = 3 SV = 4	83	E1BQC2_CHICK	0	34	43	0	30	37
**8**	Ovalbumin OS = *Gallus gallus* OX = 9031 GN = SERPINB14 PE = 1 SV = 2	43	OVAL_CHICK (+1)	0	9	17	0	3	6

## Data Availability

Not applicable.

## Abbreviations

AOAC	Association of Official Agricultural Chemists
ES MS/MS	Electrospray mass spectrometry
G1c	Granule fraction generated after the centrifugation of egg yolk
G1p	Granule fraction generated after pressurization of G1c
G2c	Granule fraction generated after the centrifugation of G1c
G2p	Granule fraction generated after the centrifugation of G1p
HDL	high-density lipoproteins
ICP-OES	Inductively coupled plasma optical emission spectroscopy
LDL	low-density lipoproteins
NanoLC	Nanoscale capillary liquid chromatography
PAGE	Polyacrylamide gel electrophoresis
P2c	Plasma fraction generated after the centrifugation of G1c
P2p	Plasma fraction generated after the centrifugation of G1p
RP	Reversed-phase
SDS	Sodium dodecyl sulphate
TEM	Transmission electron microscopy
TSC	Total spectrum count
UHPH	Ultra-high pressure homogenization
VTG	Vitellogenin
